# Exploring the Change of Host and Microorganism in Chronic Obstructive Pulmonary Disease Patients Based on Metagenomic and Metatranscriptomic Sequencing

**DOI:** 10.3389/fmicb.2022.818281

**Published:** 2022-03-16

**Authors:** Jing Yang, Qiang Zhang, Jun Zhang, Yan Ouyang, Zepeng Sun, Xinlong Liu, Feng Qaio, Li-Qun Xu, Yunfei Niu, Jian Li

**Affiliations:** ^1^The Key Laboratory of Developmental Genes and Human Disease, School of Life Sciences and Technology, Southeast University, Nanjing, China; ^2^Department of Respirology, Zhongda Hospital, Southeast University, Nanjing, China; ^3^Department of Respirology, The Fourth Affiliated Hospital of Nanjing Medical University, Nanjing, China; ^4^Vision Medicals, Guangzhou, China; ^5^China Mobile (Chengdu) Industrial Research Institute, Chengdu, China

**Keywords:** COPD, metatranscriptome, metagenome, immune, macrophage

## Abstract

**Background:**

Chronic obstructive pulmonary disease (COPD) is a universal respiratory disease resulting from the complex interactions between genes and environmental conditions. The process of COPD is deteriorated by repeated episodes of exacerbations, which are the primary reason for COPD-related morbidity and mortality. Bacterial pathogens are commonly identified in patients’ respiratory tracts both in the stable state and during acute exacerbations, with significant changes in the prevalence of airway bacteria occurring during acute exacerbation of chronic obstructive pulmonary disease (AECOPD). Therefore, the changes in microbial composition and host inflammatory responses will be necessary to investigate the mechanistic link between the airway microbiome and chronic pulmonary inflammation in COPD patients.

**Methods:**

We performed metatranscriptomic and metagenomic sequencing on sputum samples for twelve AECOPD patients before treatment and for four of them stable COPD (stabilization of AECOPD patients after treatment). Sequencing reads were classified by Kraken2, and the host gene expression was analyzed by Hisat2 and HTseq. The correlation between genes was obtained by the Spearman correlation coefficient. Mann–Whitney *U*-test was applied to identify microbes that exhibit significantly different distribution in two groups.

**Results:**

At the phyla level, the top 5 dominant phyla were *Firmicutes*, *Actinobacteria*, *Proteobacteria*, *Bacteroidetes*, and *Fusobacteria*. The proportion of dominant gates in metagenomic data was similar in metatranscriptomic data. There were significant differences in the abundance of specific microorganisms at the class level between the two methods. No significant difference between AECOPD and stable COPD was found. However, the different expression levels of 5 host genes were significantly increased in stable COPD and were involved in immune response and inflammatory pathways, which were associated with macrophages.

**Conclusion:**

Our study may provide a clue to investigate the mechanism of COPD and potential biomarkers in clinical diagnosis and treatment.

## Introduction

Chronic obstructive pulmonary disease (COPD) is a prevalent respiratory disease resulting from the complex interaction between genes and the environment (Agustí and Vestbo, 2011). Although genetics may play a significant role in COPD progression, the infection, smoking and environmental condition may also be other risk factors involved (Hansel et al., 2016; Yang et al., 2021). COPD is one of the serious public health problems with high incidence and heavy social and economic burden. The Chinese Lung Health Study published a manuscript in The Lancet in 2018 that COPD is highly prevalent in the Chinese adult population (Wang et al., 2018). Meanwhile, the process of COPD deteriorates due to repeated episodes of exacerbations (Pavord et al., 2016; Halpin et al., 2017), even after formal treatment, a significant number of patients suffer repeated acute exacerbations and even death (Ritchie and Wedzicha, 2020). Thus, there is an urgent need to find biomarkers of acute exacerbation in COPD patients, provide early warning for clinical treatment, and find effective treatment measures to prevent acute exacerbation leading to the rapid decline in lung function.

Currently the incidence of COPD is related to a variety of factors: smoking, external environment pollution and infection. Infection is the main risk factors leading to the incidence of COPD, resulting in the pulmonary inflammatory response and other changes (Cho et al., 2019; Yang et al., 2021). Researchers observe that healthy people still have lung microorganisms, and the changes of microorganisms can cause lung disease (Moffatt and Cookson, 2017). In the clinic, it is challenging to find pathogens accurately using conventional etiological diagnostic techniques. The contribution of the microbiome to COPD pathology and the potential of clinical microbiome biomarkers in COPD are still in the early stages of research (Ditz et al., 2020). With advanced technologies, microbiome studies have moved from 16S rRNA gene studies to complete genome and transcriptome sequencing and metabolome characterization. Metagenomics and metatranscriptomics, a new generation of sequencing technologies, can reveal the high-throughput, unbiased determination of the composition of pathogens associated with COPD and the co-expression of pathogens and host genes. Thus, changes in the microorganisms associated with COPD status were revealed, and essential clues were provided for further study of microorganisms and COPD progression. Sputum is a non-invasive and readily available biological sample that can provide much disease information about COPD and has become the preferred sample type for microbiome studies. Although the sputum microorganisms may only partially reflect the respiratory microbiome, there is increasing evidence that microbial community structure and diversity are associated with disease severity in both stable COPD and acute exacerbations (Sun et al., 2020).

Additionally, many of studies on the identification and classification of pathogenic microorganisms are still controversial. It has been suggested that microorganisms are commonly detected in patients’ respiratory tracts both in the stable state and during acute exacerbations (Garcha et al., 2012). Microbial colonization may also play a role in stable disease (Leung et al., 2017). Respiratory bacterial and viral infections are important triggers of AECOPD (Leung et al., 2017; Yin et al., 2018). Miravitlles (2002) proposed the “bacterial threshold hypothesis” that there is bacterial colonization in the airway of COPD patients with a small amount of stable period. But it is not enough to cause acute exacerbation, when some endogenous or exogenous factors increase the bacterial load, it will produce airway inflammation and induce acute exacerbation when the bacterial load exceeds a certain threshold. There are some studies revealing that microorganisms should be responsible for the exacerbation of COPD. However, studies in the past found that bacterial pathogens were isolated from sputum at the same rate during acute exacerbation and stabilization (Sethi et al., 2007). Meanwhile, a study has confirmed that new pathogenic strains of stable colonization bacteria can be detected from AECOPD patients. It is believed that AECOPD may not be caused by the original plant bacteria but by the acquisition of new pathogenic strains or the change of bacterial antigen determinant (Sethi et al., 2002). Therefore, bacterial pathogens are commonly identified in patients’ respiratory tracts both in the stable state and during acute exacerbations, with significant changes in the prevalence of airway bacteria occurring during acute exacerbations of COPD (Garcha et al., 2012; Han et al., 2012). However, Sethi et al. (2007) found no difference in bacterial concentrations between AECOPD and stable COPD or lower bacterial concentrations in some cases, suggesting that changes in bacterial load may unlikely be an important mechanism of disease exacerbation. Concentrations of the new strains increased during exacerbations compared to the new strains, but these differences were within 1 log (10 times). These differences in bacterial load may reflect the result of host-pathogen interactions rather than independent mechanisms.

Recently, airway microbial environment and the immune system also have become the focus in the pathogenesis of COPD. The shifts in airway microbial composition can drive specific inflammatory responses, such as Th17-mediated inflammation, which is known to function in antibacterial immunity (Segal et al., 2016; Yadava et al., 2016). Studies integrating perturbations in microbial composition with host inflammatory responses will be necessary to develop a mechanistic link between the airway microbiome and chronic pulmonary inflammation in COPD patients. Lymphocytes and neutrophils play crucial role in the immune response; impaired immunity may lead to low lymphocyte count, which further increases the risk of respiratory infection, eventually influence the development of COPD (Huang et al., 2020; D’Anna et al., 2021). Pulmonary macrophages (LMs) are primarily found in the airways and lung tissues, and phenotypic changes are associated with chronic inflammatory responses and disease progression in various chronic lung diseases, including COPD (Arora et al., 2018). In response to injury, macrophages rapidly change their behavior from supporting inflammation (often called M1) in the early stages of healing to a state that promotes inflammation and healing (often called M2) in the later stage (Arora et al., 2018). The number of macrophages in the sputum of patients with COPD increased significantly, while M2 macrophages dominated the bright areas of COPD lung tissue (Yamasaki and Eeden, 2018). Two major subtypes of M2 macrophages are involved in wound healing, namely those stimulated (at least *in vitro*) by interleukin-4 (IL-4) (called M2a) and those stimulated by IL-10 (called M2c) (Lurier et al., 2017). M2c macrophages may play a role in the early stages of wound healing. Many M2c specific genes have been identified to be involved in angiogenesis, matrix modification, phage formation. And M2c related genes are up-regulated early after injury (Ohta et al., 2016).

Furthermore, the interaction between bacteria and host is quite complex, and inflammation plays a central role in the occurrence and development of COPD (Caverly et al., 2019). Host factors such as proteases and oxidants have been known to promote tissue damage and amplify inflammatory processes. Bacterial factors such as oligosaccharides, surface proteins and proteases may damage lung tissues directly or indirectly by promoting the host inflammatory response (Abusriwil and Stockley, 2007). Abnormal inflammatory response and impaired airway immune system provide an opportunity platform for bacterial colonization and infection, leading to a “vicious cycle” (Su et al., 2018). Some studies have found the interaction between the microbiome and the host through multi-omic meta-analysis, the correlation relationship of “microbiome-metabolite-host” has been proposed (Wang et al., 2020). Therefore, we presumed that the frequent exacerbation might be related to the change of host. However, there are few reports on host gene changes and their correlation with the inflammatory state.

By using metagenomic and metatranscriptomic approaches, the dynamic changes in microorganisms and host genes expression between stable COPD and acute exacerbation can be clarified. To provide a new diagnosis and treatment idea for controlling the disease progression of COPD, we explore the changes in the abundance of microorganisms and the different expression levels of host genes between AECOPD and stable COPD.

## Materials and Methods

### Subjects and Clinical Samples

This prospective observational study evaluated adults with AECOPD admitted to the Southeast University Zhongda Hospital and the fourth Affiliated Hospital of Nanjing Medical University. Twelve patients with AECOPD before treatment and four of them stable COPD (stabilization of AECOPD patients after treatment) were enrolled in this study. These stable patients were reviewed two months after discharge. Meanwhile, all AECOPD subjects were male patients with a smoking index over 400, and they had the ability to expectorate and be received stable follow-up. Patients could receive regular drug control after discharge. The exclusion conditions included bronchial asthma and allergic rhinitis, female and non-smoking male patients, imaging patients with obvious structural lung disease, such as bronchitis, pulmonary tuberculosis, pulmonary fibrosis, patients with obvious immune dysfunction or long-term use of immunosuppressive drug. Clinical information was obtained for each enrolled patient (Supplementary Table 1). This study was approved by the Ethics Committee of Zhongda Hospital (Number: 2020ZDSYLL121-P01).

The 16 Sputum samples were classified according to acute exacerbations cases (≥2 moderate or severe exacerbations or ≥1 hospitalization for AECOPD, *n* = 12) and stable COPD (stabilization of AECOPD patients after treatment, COPD, *n* = 4). The sputum samples were collected from patients during the acute exacerbations and the stable state. All participants reported that they had not used antiseptic mouthwash before sample collection and expectorated unstimulated 3 to 5 ml Sputum into Sputum box, which was immediately stored at −80°C.

### Metagenomic and Metatranscriptomic Sequencing

DNA and RNA concentrations were measured using a Qubit Fluorometer (Thermo Fisher Scientific, Carlsbad, CA, United States). RNA and DNA were extracted from 600 μL of patient sputum using the special kit (Vision Medicals, China). RNA was reversely transcribed to generate cDNA by ligating with T4 ligase and N6 random primers. DNA was added adapter and barcode using the Nextera library preparation kit (Illumina). The final RNA-seq and DNA-seq libraries underwent a 50 bp single-end on Illumina Nextseq 550.

### Metagenomic Sequencing and Bioinformatic Analysis

Sequencing reads were processed, the reads were inspected by FastQC and filtered by Trimmomatic (Bolger et al., 2014). Low-quality reads were filtered, high-quality reads were retained for subsequent analysis. All filtered reads that could be appropriately mapped to the human reference genome (GRCh38) or human genes sequences (Ensembl release 83) by Bowtie2 (v2.2.6) were suspected to represent host contamination and were discarded from further analysis (Langmead and Salzberg, 2012). The taxonomic classification was performed using Kraken2 (Wood et al., 2019) against the NCBI Ref gene database.

### Metatranscriptomic Sequencing and Bioinformatic Analysis

The raw data were filtered by Trimmomatic, and the filtered reads were aligned with the human genome (NCBI GRC h38) using Hitsat2 and HTseq. The filtered data was classified species by Kraken2. Furthermore, human genes count data were analyzed using the Bioconductor package Limma (version 3.50) in R (version 4.0.1) statistical programming environment. We limited our differential expression analysis to the derivation cohort of 4 AECOPD and 4 stable COPD samples to avoid batch-related confounding and class imbalance. Differentially expressed genes with *P* < 0.01 and |LogFC| > 1 were used to evaluate. The correlation analysis of the differentially expressed genes, adopting Spearman’s correlation coefficient, was performed in R. We constructed a network of the differentially expressed genes with AECOPD and stable COPD. The network was generated using STRING and Cytoscape (v3.8.2).

### Statistical Analyses

The Mann–Whitney *U*-test followed by Bonferroni correction was used to test for significant differences in taxonomic levels between groups. The Bonferroni-adjusted *p*-Values were calculated as 0.05 divided by the numbers of parameters in table.

## Results

### Overview of Demographic Data

To compare the changes of AECOPD patients and stable COPD patients, sputum specimens were collected from 12 AECOPD patients during exacerbation visits and four stable COPD patients (stabilization of AECOPD patients after treatment). The characteristics of patients are summarized in Table 1. The patients’ age ranges from 59 to 92 years, with a mean BMI ranging from 18.37 to 30.85 kg/m^2^. All patients were male. None of the patients had received antibiotics before sample collection. Furthermore, eosinophile and basophile counts decreased in stable COPD patients. There were no consistent changes in neutrophil and lymphocyte counts, likely due to individual circumstances and differences in immunity (Supplementary Table 1).

**TABLE 1:** Background information for the acute exacerbation of chronic obstructive pulmonary disease (AECOPD) and stable chronic obstructive pulmonary disease (COPD) patients.

Characteristics	AECOPD		stable COPD
Number of patients	8	4	4
**Age (years)**			
Range	59-92	65-86	
Mean ± S	73.25 ± 10.82	73.75 ± 8.81	
**Current smoking**			
No	5 (62.50%)	3 (75%)	
Yes	3 (37.50%)	1 (25%)	
**BMI (kg/m2)**			
Range	20.70-30.85	18.37-25.69	
Mean ± S	24.53 ± 4.49	22.26 ± 3.32	
**Lymphocyte%**			
Range	7.10-40.70	11.00-18.00	7.50-19.30
Mean ± S	19.36 ± 11.14	13.80 ± 3.00	12.17 ± 6.27
**Neutrophils%**			
Range	58.00-87.20	64.90-77.10	69.50-88.80
Mean ± S	74.49 ± 10.26	73.50 ± 5.76	81.27 ± 10.32
**Eosinophile%**			
Range	0-2.80	2.70-6.70	0.10-0.90
Mean ± S	0.80 ± 1.14	4.33 ± 1.69	0.60 ± 0.44
**Basophile%**			
Range	0-0.70	0.40-0.90	0.10-0.30
Mean ± S	0.29 ± 0.27	0.60 ± 0.22	0.17 ± 0.12

### Compositional Profiles of the Metagenomic and Metatranscriptomic Data

**FIGURE 1:**
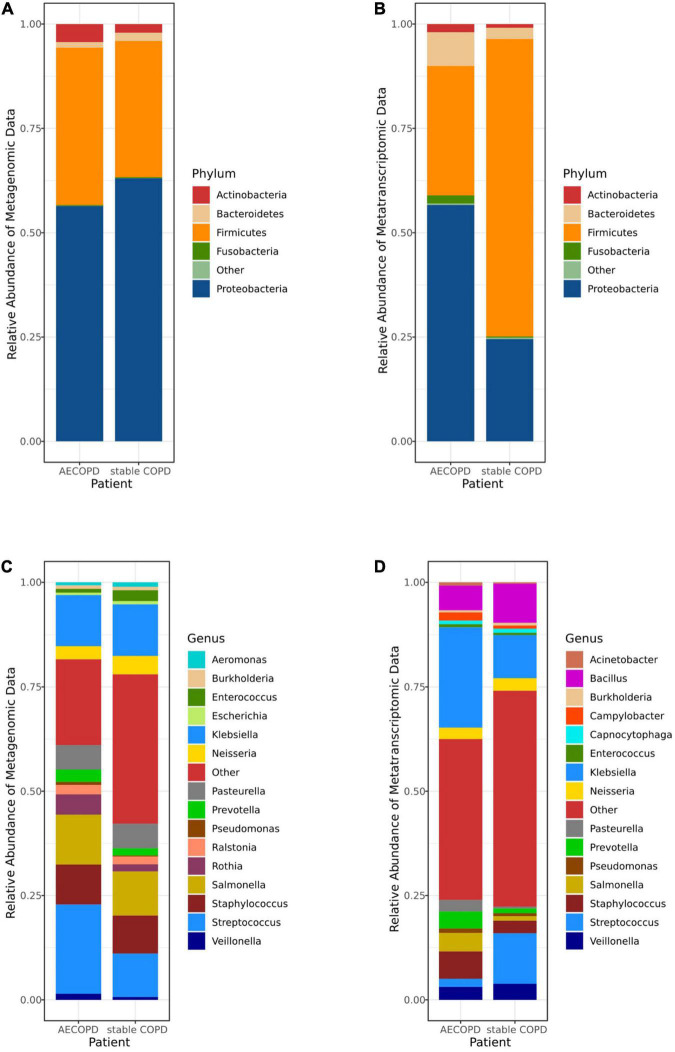
The microbiomes compositional profiles of the Acute Exacerbation of Chronic Obstructive Pulmonary Disease (AECOPD) patients and stable COPD (stabilization of AECOPD patients after treatment) patients. The stacked bar represents differentially relative abundance of microorganisms in the AECOPD vs. stable COPD groups. **(A,B)** shows the relative abundance of the most dominant taxa at the phylum levels in metagenomic data **(A)** and metatranscriptomic data **(B)**. **(C,D)** shows the most dominant taxa distributions at the genus levels of the sputum microbiomes in metagenomic data **(C)** and metatranscriptomic data **(D)**.

Sequencing reads were inspected and filtered, and the high-quality reads were retained for subsequent analysis. The number of reads varied between 9,736,769 and 38,402,159 reads per sample in metagenomic data and from 12,831,519 to 59,732,739 in metatranscriptomic data. In metagenomic data, 5 phyla (*Firmicutes*, *Actinobacteria*, *Proteobacteria*, *Bacteroidetes* and *Fusobacteria*) were detected in the samples (Figure 1A), 10 classes (*Actinomycetia*, *Bacteroidia*, *Flavobacteriia*, *Bacilli*, *Clostridia*, *Negativicutes*, *Fusobacteriia*, *Alphaproteobacteria*, *Betaproteobacteria*, *Gammaproteobacteria*) were the most abundant (Supplementary Figure 1A). Among all genera, *Enterococcus*, *Ralstonia*, *Prevotella*, *Rothia*, *Neisseria*, *Pasteurella*, *Staphylococcus*, *Salmonella*, *Klebsiella*, *Streptococcus* were highly diverse, respectively (Figure 1C). The Order and Family were detected in different groups (Supplementary Table 2). In metatranscriptomic data, at the phyla level, *Firmicutes*, *Actinobacteria*, *Proteobacteria*, *Bacteroidetes* and *Fusobacteria* were the most abundant in the samples (Figure 1B), 10 classes (*Gammaproteobacteria*, *Bacilli*, *Betaproteobacteria*, *Bacteroidia*, *Negativicutes*, *Fusobacteriia*, *Actinomycetia*, *Epsilonproteobacteria*, *Flavobacteria*, *Alphaproteobacteria*) were most diverse (Supplementary Figure 1B). The top 15 most abundant genera (*Klebsiella*, *Bacillus*, *Salmonella*, *Prevotella*, *Staphylococcus*, *Veillonella*, *Streptococcus*, *Neisseria, Burkholderia*, *Campylobacter*, *Pasteurella*, *Pseudomonas*, *Capnocytophaga*, *Enterococcus* and *Acinetobacter*) were detected in the samples. The relative abundance of the dominant Order (Supplementary Figures 1C,D) and Family (Supplementary Figures 1E,F) were detected in different groups (Figure 1D and Supplementary Table 3).

### Comparison of Microbiome Composition Between Metatranscriptomic Data and Metagenomic Data

**FIGURE 2:**
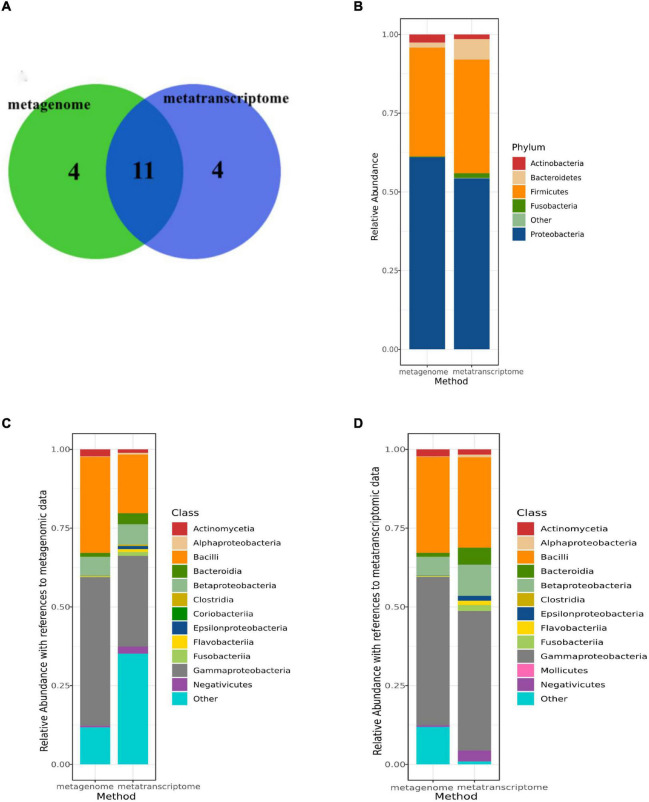
Comparison of microbiome composition between metatranscriptomic data and metagenomic data. **(A)** Overlap of identified genera between two data sets for the 12 AECOPD patients and 4 stable COPD patient (metatranscriptomic data and metagenomic data). **(B)** The read abundance of the top 5 most abundant phylum in two data sets (metatranscriptomic data and metagenomic data). **(C)** The read abundance of the top 12 most abundant classes in metagenomic data in two data sets (metatranscriptomic data and metagenomic data). **(D)** The read abundance of the top 12 most abundant classes in metatranscriptomic data in two data sets (metatranscriptomic data and metagenomic data).

In Figure 2A, the colors represent different methods and the overlap represents equal microorganisms (metagenomic data and metatranscriptomic data). Among the microorganism detected in all patients, we first compared the bacterial of the sputum microorganism in different method groups (metagenomic data and metatranscriptomic data). At the phylum level, the top 5 dominant phyla were *Firmicutes*, *Actinobacteria*, *Proteobacteria*, *Bacteroidetes* and *Fusobacteria*, and the proportions of the dominant phyla were similar in the metagenomic data vs. metatranscriptomic data (Figure 2B). However, a significant difference in the abundance of specific microbes at the class level was observed among the two methods. By comparing them, we found that the same microorganism was identified in the different abundances in two types of data (Figures 2C,D). For example, *Coriobacteria* had a higher abundance in the metagenomic data, while *Mollicutes* were more highly enriched in the metatranscriptomic data.

### Differences in the Abundance of Bacteria From Patients With Acute Exacerbation of Chronic Obstructive Pulmonary Disease and Stable Chronic Obstructive Pulmonary Disease Patients

**FIGURE 3:**
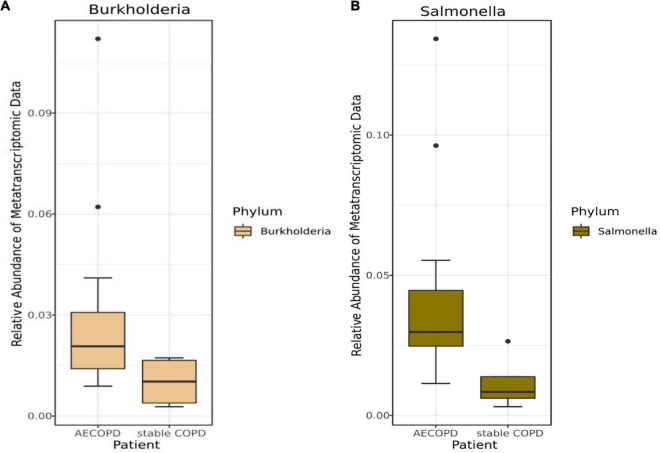
Differences in the abundance of bacteria at the genus levels in the metatranscriptomic data with AECOPD and stable COPD patients. The box and whisker plots show the relative abundance of *Burkholderia* **(A)** and *Salmonella* **(B)** in the AECOPD and vs. stable COPD.

Mann–Whitney *U*-test result showed there was no significant difference between patients with AECOPD and stable COPD (Supplementary Tables 4, 5). Various difference was observed in patients with AECOPD compared with those in the other group, and neither of the differences was statistically significant (*p* > 0.00083) in the metagenomic data (Supplementary Table 4). Furthermore, similar results were obtained from the analysis of the metatranscriptome data (Supplementary Table 5). Meanwhile, among the top 15 genera, the relative abundance of *Salmonella* (*p* = 0.02) and *Burkholderia* (*p* = 0.10) was decreased in the metatranscriptomic data but not significantly different in the stable COPD patients compared with that in the AECOPD patients (Figure 3 and Supplementary Table 5).

### Differentially Expressed Genes in Acute Exacerbation of Chronic Obstructive Pulmonary Disease and Stable Chronic Obstructive Pulmonary Disease Patients and the Correlation Between Genes

**FIGURE 4:**
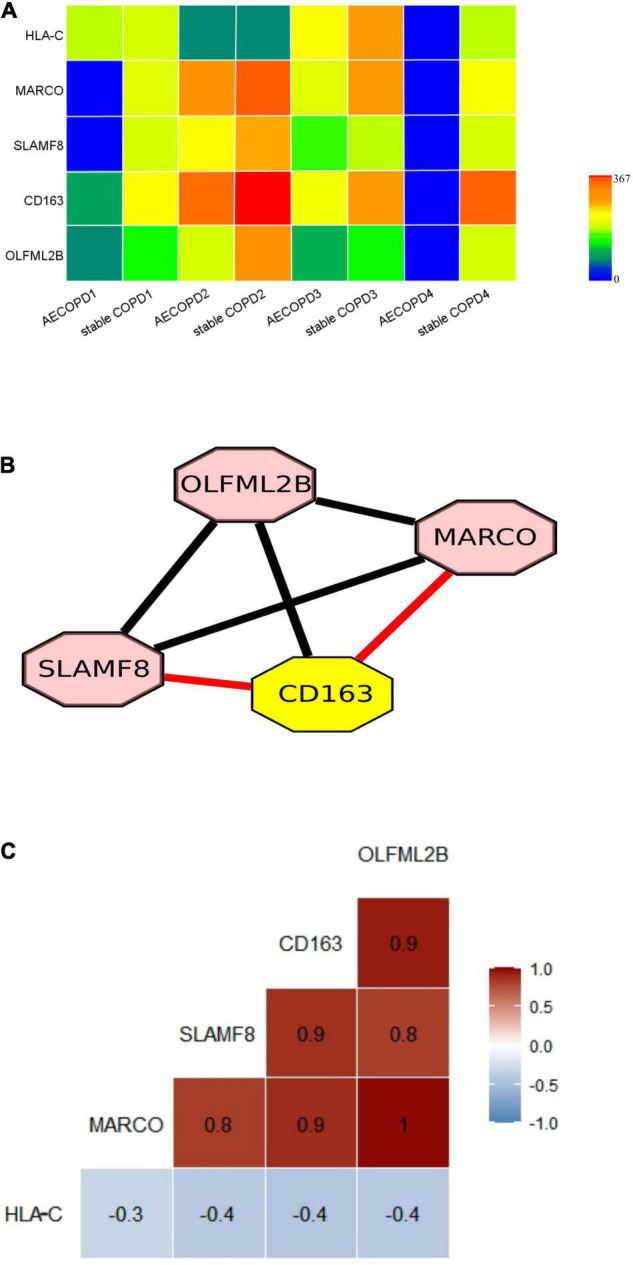
Differentially expressed genes in AECOPD and stable COPD patients and the correlation between genes. **(A)** Gene expression of 5 key genes. Gene expression levels were calculated as log2 (normalized number of transcripts per million [TPM] + 0.00001). **(B)** The nodes represent genes. The networks of the 5 genes were built using Cytoscape. (The red line indicates that the genes are co-expression, and the result is consistent with STRING, the black line indicates that they just have correlation in Spearman coefficient). **(C)** The figure show Spearman correlation coefficients between the 5 genes.

A previous study presenting no significant difference was found at the genus level between patients with AECOPD and stable COPD in the metagenomic data and metatranscriptomic data. A significant proportion of the metatranscriptomic data in the sputum samples were derived from human cells (Collins et al., 2014), enabling us to investigate the host-microbe interaction. By comparison, we found that the expression levels of 5 genes (*P* < 0.01 and |LogFC| > 1) (Figure 4A and Supplementary Table 6). The significantly different expressed genes were analyzed by DAVID. The result showed that the genes were enriched in immune response and inflammatory pathways. Specifically, they are all involved with macrophages (Table 2).

**TABLE 2:** The Annotation of different expression genes.

Gene Name	Annotation	GO
CD163	acute-phase response; inflammatory response	0006953
SLAMF8	integral component of membrane; immunoglobulin like domain	0016021
MARCO	innate immune response;	0045087
HLA-C	immune response	0006955
OLFML2B	proteinaceous extracellular matrix	005578

To explore the potential genetic co-existence and co-exclusion relationships, we performed an interaction network analysis. The specific network was built and estimated based on the expression of the gene using Cytoscape (Figure 4B), STRING and Spearman correlation coefficient. Each node represents a gene. All plotted nodes of the networks with significant coefficients are shown in Figure 4B. In total, CD163 was included in a closed correlated network containing MARCO, SLAMF8 and the Spearman correlation coefficients between the 5 genes in the groups (Figure 4C, Table 3, and Supplementary Table 7).

**TABLE 3:** The correlation between the genes.

Gene1	Gene2	Correlation coefficient
MARCO	SLAMF8	0.82
MARCO	CD163	0.89
MARCO	OLFML2B	0.97
SLAMF8	CD163	0.87
SLAMF8	OLFML2B	0.85
CD163	OLFML2B	0.94

*Table of the Spearman correlation from the AECOPD and stable COPD (stabilization of AECOPD patients after treatment) patients with the 5 genes.*

## Discussion

The most crucial finding of this study was no significant difference in the abundance of microorganisms at every level between AECOPD and stable COPD, but the different expression levels of 5 host genes significantly increased in stable COPD, which was related to the immune system. In our study, we performed metatranscriptomic and metagenomic sequencing on sputum samples to analyze the differences in the pulmonary microbiome in patients with AECOPD and stable COPD. In the analysis of the metagenomic data, it was found that *Proteobacteria* and *Firmicutes* were the first and second most abundant phylum in samples. However, it has been shown that there are differences in microbiota at different locations in the respiratory tract (Dickson et al., 2016). The sputum-induced microbiota may represent the lower airway microbiota, which would explain differences in the composition of the microbiota in BALF or lung samples. Pragman et al. (2018) analyzed the microbiota in oral, bronchial and lung tissue samples from individual patients and found that oral bacteria were true members of the lung microbiota in early COPD and showed ecological drift. The same result was obtained for metatranscriptomic analysis. After comparison, it was found that the pulmonary microbiome of patients with severe COPD and stable COPD was consistent at the phylum level, and differences appeared from class to class.

Interestingly, we found that most of the microorganisms detected in AECOPD patients and stable COPD patients were common respiratory microorganisms, and there was no significant difference between AECOPD and stable COPD. The top 15 genera in sputum samples from patients with AECOPD were mainly common genera. In addition, the relative abundance of *Salmonella* and *Burkholderia* were decreased in with stable COPD patients compared with AECOPD patients, but it was not statistically significant. In earlier studies, bacterial pathogens were isolated from sputum at the same rate during acute exacerbations and stable periods, but strains within bacterial species were not distinguished. Meanwhile, Sethi et al. (2007) found no difference in bacterial concentration between AECOPD and stable phase, suggesting that changes in bacterial load may unlikely be an essential mechanism of disease exacerbation. Therefore, we further explored the changes in the host of AECOPD patients and stable COPD patient.

In our study, heavy-duty patients discharge 2 months of the bacteria in the abundance of no significant difference of change, but through the analysis of metatranscriptomic data, we found that the host genes significantly increased (*p* < 0.01) and were involved in the immune response. The infection triggers NK activation, which may be particularly related to HLA-C molecules. The phenotype of tumor-associated macrophages in lung cancer is characterized by M2 class markers, such as CD163, CD204, and MARCO, which are transmembrane receptors (Lurier et al., 2017). Our results showed that MARCO and CD163 gene expression were significantly increased in stable COPD, which was consistent with previous studies. Both MARCO and CD163 are M2c specific genes with similar expression patterns (Ohta et al., 2016). The significantly decreased expression of MARCO and CD163 may cause impaired inflammatory control associated with early lung injury in COPD (Liu et al., 2020). We found that the expression level of MARCO and CD163 increased, suggesting that lung injury in patients was reduced in stable COPD. Meanwhile, studies have shown that both CD163 and MARCO play potential roles in M2c macrophages and are up-regulated early after injury, and their expressions are positively correlated (Ohta et al., 2016), which is consistent with our research results. SLAMF8 is mainly expressed in macrophages and it regulates the development and function of many immune cells (Zeng et al., 2020). Our results showed that SLAMF8 expression increased in stable COPD patient, which can be used to alleviate inflammatory conditions in patients aggravated by infection (Wang et al., 2015; Zou et al., 2019).

A significant limitation of our study is that we did not conduct a large sample study. Further studies of longitudinal sampling of each individual at the time point of deterioration and stabilization after treatment are essential for monitoring microbiome dynamics, clinical phenotype and treatment response, but further validation is needed as our sample size can only provide guesses and clues. Another limitation of our study is that clinical testing of macrophages was not performed. We observed that the changes in genes were all related to macrophages. Genes changes were aligned with some previous research (Wang et al., 2015; Ohta et al., 2016; Zou et al., 2019; Liu et al., 2020; Zeng et al., 2020), but the clinical test data were not analyzed. In this study, we excluded the influence of gender, smoking and other factors on microbes, focusing mainly on analyzing the status of male smokers and finding the phenomenon of immune abnormalities. In the follow-up study, we will systematically analyze the situation of more types of patients as well as the changes of specific immune components.

## Conclusion

We explored that the sputum transcriptionally active microbiome changed in patients with AECOPD and stable COPD. Additionally, the differentially expressed genes between AECOPD and stable COPD were found. These findings provide clues for further studies to investigate the progress of COPD and potential biomarkers in clinical diagnosis.

## Data Availability Statement

The data presented in the study are deposited in the https://ngdc.cncb.ac.cn/gsa-human/browse/HRA001944 repository, accession number HRA001944.

## Ethics Statement

The studies involving human participants were reviewed and approved by the Ethics Committee of Zhongda Hospital. The patients/participants provided their written informed consent to participate in this study. The animal study was reviewed and approved by the Ethics Committee of Zhongda Hospital.

## Author Contributions

JY carried out the data analysis and manuscript draft. QZ collected the samples and clinical trials and reviewed the manuscript. JZ collected the samples. YO carried out the data analysis. ZS, XL, and FQ drafted the manuscript. L-QX reviewed the manuscript. YN carried out the overall project conceptualization. JL carried out the overall project design, conceptualization, investigation, supervision, acquisition of funding and resources, and reviewing of the manuscript. All authors contributed to the article and approved the submitted version.

## Conflict of Interest

YO and YN were employed by company Vision Medicals. The remaining authors declare that the research was conducted in the absence of any commercial or financial relationships that could be construed as a potential conflict of interest.

## Publisher’s Note

All claims expressed in this article are solely those of the authors and do not necessarily represent those of their affiliated organizations, or those of the publisher, the editors and the reviewers. Any product that may be evaluated in this article, or claim that may be made by its manufacturer, is not guaranteed or endorsed by the publisher.

## Supplementary Material

Supplementary Figure 1 The microbiomes compositional profiles of the AECOPD patients and stable COPD (stabilization of AECOPD patients after treatment) patients. The stacked bar represents differentially relative abundance of microorganisms in the AECOPD vs. stable COPD groups. **(A,B)** shows the relative abundance of the most dominant taxa at the class levels in metagenomic data **(A)** and metatranscriptomic data **(B)**. **(C,D)** shows the most dominant taxa distributions at the order levels of the sputum microbiomes in metagenomic data and metatranscriptomic data. **(E,F)** shows the most dominant taxa distributions at the family levels of the sputum microbiomes in metagenomic data and metatranscriptomic data.
