# Supplementary figures and images for: Exploring the Change of Host and Microorganism in Chronic Obstructive Pulmonary Disease Patients Based on Metagenomic and Metatranscriptomic Sequencing
Source: Front Microbiol. 2022 Mar 16;13:818281. doi: 10.3389/fmicb.2022.818281 (PMC8966909; doi:10.3389/fmicb.2022.818281)

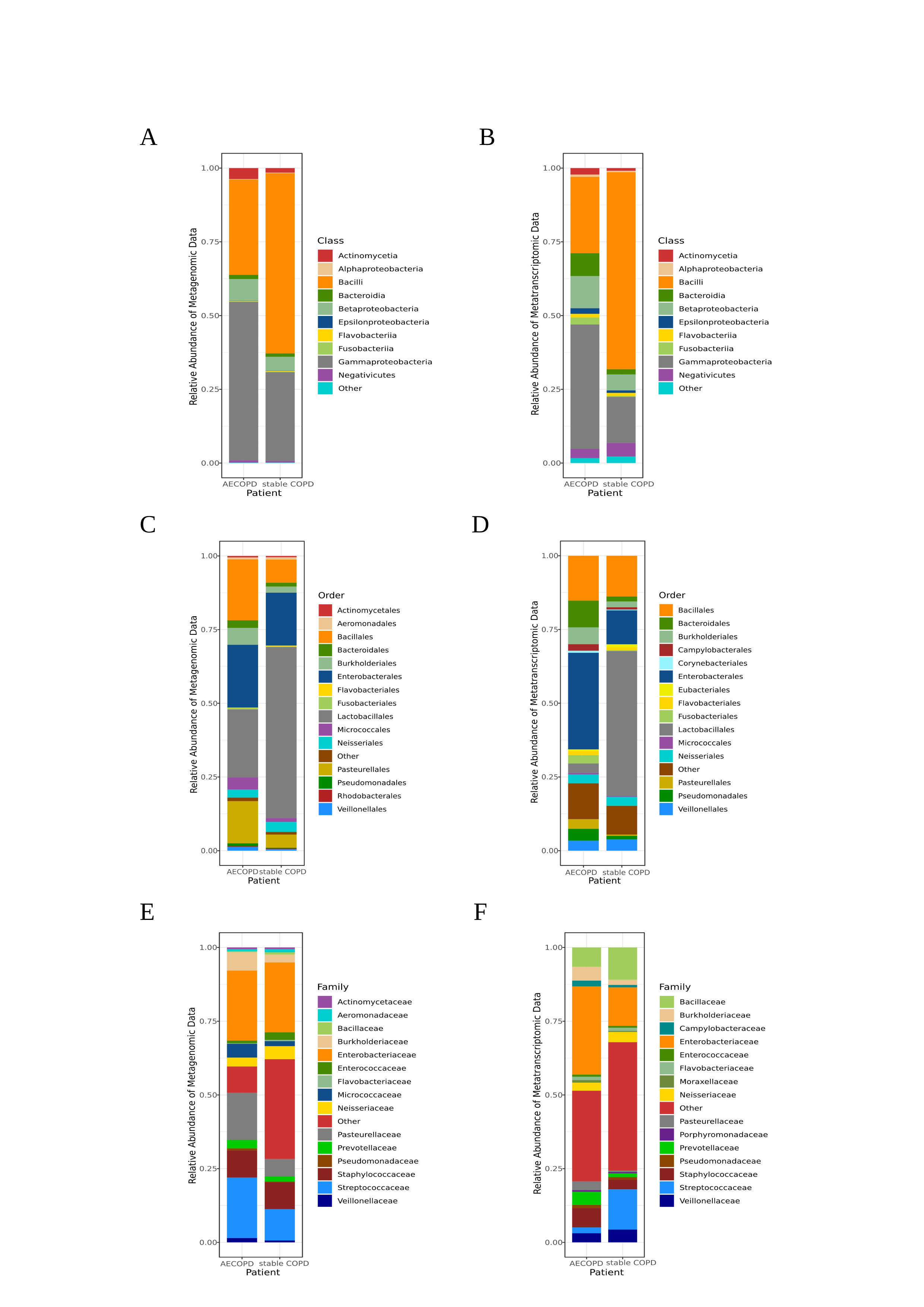

Supplement: Supplementary Figure 1 — The microbiomes compositional profiles of the AECOPD patients and stable COPD (stabilization of AECOPD patients after treatment) patients. The stacked bar represents differentially relative abundance of microorganisms in the AECOPD vs. stable COPD groups. (A,B) shows the relative abundance of the most dominant taxa at the class levels in metagenomic data (A) and metatranscriptomic data (B). (C,D) shows the most dominant taxa distributions at the order levels of the sputum microbiomes in metagenomic data and metatranscriptomic data. (E,F) shows the most dominant taxa distributions at the family levels of the sputum microbiomes in metagenomic data and metatranscriptomic data. [file Data_Sheet_1.zip › Supplementary_Material/Figure S1.JPEG]
